# Are the Effects of Response Inhibition on Gambling Long-Lasting?

**DOI:** 10.1371/journal.pone.0070155

**Published:** 2013-07-26

**Authors:** Frederick Verbruggen, Rachel C. Adams, Felice van ‘t Wout, Tobias Stevens, Ian P. L. McLaren, Christopher D. Chambers

**Affiliations:** 1 Psychology, College of Life and Environmental Sciences, University of Exeter, Exeter, United Kingdom; 2 School of Psychology, Cardiff University, Cardiff, United Kingdom; Universidad de Granada, Spain

## Abstract

A recent study has shown that short-term training in response inhibition can make people more cautious for up to two hours when making decisions. However, the longevity of such training effects is unclear. In this study we tested whether training in the stop-signal paradigm reduces risky gambling when the training and gambling task are separated by 24 hours. Two independent experiments revealed that the aftereffects of stop-signal training are negligible after 24 hours. This was supported by Bayes factors that provided strong support for the null hypothesis. These findings indicate the need to better optimise the parameters of inhibition training to achieve clinical efficacy, potentially by strengthening automatic associations between specific stimuli and stopping.

## Introduction

Convergent clinical evidence suggests that executive control in the motor domain might share mechanisms with high-level decision-making. Poor response inhibition during adolescence predicts later substance dependence [1], and response-inhibition deficits have been observed in impulse-control disorders [2], [3], such as attention deficit/hyperactivity disorder [4], substance abuse disorders [5], [6], and gambling disorders [7]–[10]; but see also [11]. More generally, problem gambling and substance addiction involve a shift from novelty-seeking, impulsive behaviour (acting quickly in pursuit of reward without consideration of adverse consequences) to compulsive behaviour (acting persistently with diminished regard for reward and despite adverse consequences); and response inhibition has been linked to both constructs [12], [13]. Thus, several authors have argued that response inhibition could play an important role in the development and maintenance of addictions and influence the outcome of treatments [12], [14]–[17].

Inspired by these empirical findings and the central role of response inhibition in neurobiological models of addiction, we recently examined whether asking people to stop simple movements had a causal effect on gambling [18]. To this end, we combined a stop-signal task with a ‘decision-making under uncertainty’ task that involved a certain element of risk (i.e. subjects could either win or lose points). Successful stop-signal performance requires an inhibitory control network, which includes (among other areas) the right inferior frontal gyrus, right middle frontal gyrus, pre-supplementary motor area, and basal ganglia [2], [3], [19], [20]. The right frontal areas have been linked to self-control and inhibition in multiple domains [21]–[23]. Therefore, we used a variant of the stop-signal task to explore whether motor control would transfer to monetary decision-making when gambling [18].

On every trial of our novel gambling task, participants were presented with 6 choice options. Each option was associated with a certain amount they could win; however, they were informed at the beginning of the experiment that wins were less probable for higher amounts. Healthy participants performed this task throughout the session. In some blocks (‘load’ blocks), participants had to perform a second task when an occasional signal occurred. Participants in the ‘stop’ condition attempted to stop the planned choice response, whereas participants in the ‘double-response’ condition executed a second response on trials where the signal occurred. We found that participants in the stop group not only became more cautious when executing their choice responses (as indexed by longer choice latencies), they also selected lower amounts with a higher probability of winning [p(win)] in ‘load’ blocks than in ‘no-load’ blocks in which no signals could occur [18]. In the double-response group, there was a numerical difference in the opposite direction; i.e. a tendency to select higher amounts with a lower p(win) in load blocks than in no-load blocks. We concluded that stopping-induced motor cautiousness transferred to monetary choice when gambling.

**Figure 1:**
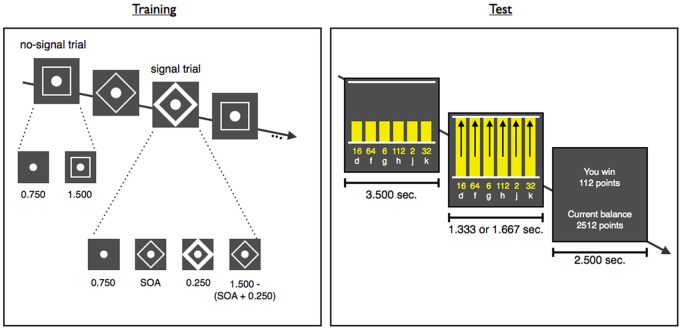
Examples of no-signal and signal trials in the training phase, and a gambling trial in the test phase (SOA = variable stimulus-onset asynchrony).

The potential overlap between control processes could open new avenues for the treatment of impulse-control disorders [22],[24]–[26]. Indeed, in two follow-up experiments, we found that training participants to stop motor responses also influenced monetary decision-making when stopping and gambling were separated in time [18]. Those experiments consisted of two phases: the training phase involved either a stop task or double-response task (Figure 1) without monetary decision-making; in the test phase, participants then performed the gambling task without an additional cognitive load. The delay between the training phase and test phase was either two minutes (Experiment 2) or two hours (Experiment 3). In both experiments, we found that participants who did the stop task preferred lower amounts with a higher p(win) in the subsequent gambling task, compared with participants who did the double-response task or participants who received no executive-control training. In sum, these results suggested that training on stopping simple motor responses could have a sustained after-effect on monetary decision-making.

In the present study, we further explored the longevity of these training effects by increasing the delay between the training phase and the test phase. In Experiment 1, the delay between the training phase and the test phase was 24 hours. In Experiment 2, we doubled the amount of training: On Days 1 and 2, participants performed the double-response or stop task, before completing the gambling task on Day 3.

## Materials and Methods

### Ethics Statement

This work was carried out at Cardiff University and the University of Exeter in compliance with ethical standards. The experiments were approved by the local research ethics committees at the School of Psychology, Cardiff University (Experiment 1) and the School of Psychology, University of Exeter (Experiment 2). Written informed consent was obtained after the nature and possible consequences of the studies were explained.

### Participants

In Experiment 1, sixty volunteers from the Cardiff University community participated for monetary compensation (£6 per hour, plus money won in the gambling task; average amount won: £1.1, range: £0–4.2). In Experiment 2, fifty-two volunteers from the University of Exeter participated for partial course credit. In each experiment, sample size, gender, age, and general levels of impulsivity (assessed by the Barratt Impulsiveness Scale –11) and risk-taking (assessed by the Stimulating-Instrumental Risk Inventory), were similar for the stop and double-response groups (Table 1). Participants were informed that they would do different tasks on consecutive days, but we did not specify the nature of the tasks in advance.

**Table 1:** Characteristics of participants included in the analyses (see the Results section for discussion of the participant exclusion criteria).

	Experiment 1		Experiment 2	
Variable	Stop	Double-response	Stop	Double-response
# participants	30	29	24	24
% female	70	66	88	75
Age	23.0	22.7	19.0	20.0
BIS-Total	64	62	60	65
BIS-Attentional	17	16	16	17
BIS-Motor	23	22	21	23
BIS-NonPlanning	24	24	23	25
SIRI-Total	38	40	38	40
SIRI-Stimulating	22	23	20	22
SIRI-Instrumental	17	18	21	18

Note: The range of possible total scores on the 11th version of the Barratt Impulsiveness Scale (BIS-11; Patton, Stanford, & Barratt, 1995) is 30 to 125; higher scores indicate more impulsive behaviour. On the Stimulating-Instrumental Risk Inventory (SIRI; Zaleskiewicz, 2001), scores of 45 and below indicate a tendency toward avoiding taking risks. Separate scores for the three BIS-and two SIRI subscales appear below the total score. Note: BIS & SIR data of four participants in Experiment 1 were missing; in Experiment 2, the BIS data of one participant was missing. Due to rounding, there are small differences between the total SIRI score and the sum of the subscales.

### Procedure

All stimuli were presented on a 19-inch LCD monitor against a grey background. The task was run using the Psychophysics Toolbox [27].

#### Training phase

Both the double-response and stop groups started with a training phase in which the primary task was to identify a go stimulus (square vs. diamond) as rapidly and accurately as possible (Figure 1). Participants responded with their left or right hands, respectively (‘C’ or ‘M’ on a keyboard).

No-signal trials started with the presentation of a fixation circle for 1.5 sec after which a white non-filled shape appeared around it (to reduce the overall duration of a session, fixation duration was 0.750 sec in Experiment 2). The shape remained on the screen for 1.5 sec and participants had to respond before it disappeared.

On signal trials, the outline of the shape turned bold after a variable delay (SOA). In Experiment 1, 25% of trials were signal trials; 33% of trials were signal trials in Experiment 2. We increased the percentage of stop signals to encourage proactive control and cautious decision-making [28]. On signal trials, participants in the stop group were instructed to refrain from responding, whereas participants in the double-response group had to press the space bar as quickly as possible with either thumb after they pressed ‘C’ or ‘M’. The SOA between the go stimulus (the shape) and signal was initially set at 0.250 sec. In the stop group, the SOA was continuously adjusted according to a tracking procedure that converged on a probability of stopping of.50; in the double-response group, we simulated a tracking procedure to produce a similar range of SOAs to the stop group (see ref. 18, for further details).

The training phase of Experiment 1 consisted of 15 blocks of 56 trials. In Experiment 2, participants completed two training sessions, each consisting of 10 blocks of 72 trials and with a 24-hour delay between them. In both experiments, participants received feedback at the end of each block: they were shown their mean RT, the number of incorrect and missed responses on no-signals, and the percentage of failed stops or double-responses (depending on the group). Participants then paused for 15 seconds before commencing the next block. In Experiment 1, participants also received immediate written feedback (presented for one second) after an error or missed response in the first block.

#### Test phase

Twenty-four hours after the training, participants completed a gambling task. On each trial, 6 vertical bars were presented next to each other; each bar was associated with a certain amount and a specific key of a keyboard (‘d’, ‘f’, ‘g’, ‘h’, ‘j’, ‘k’; Figure 1). Participants were instructed to select one of the amounts by pressing the corresponding key, and without revealing the exact probabilities, they were informed at the beginning of the experiment that p(win) was lower for higher amounts.

Each trial started with the presentation of the ‘start’ bars, amounts, and the associated keys. The bars appeared between two horizontal lines. After 3.5 sec the bars started rising together. All bars reached the top line after 1.33 sec on ‘low-bar‘ trials, and after 1.67 sec on ‘high-bar‘ trials (the distance between bottom- and top line was approximately 7.5 cm on ‘low bar’ trials & 9 cm on ‘high bar’ trials; both trial types occurred with equal probability in each block). Trials ended 0.5 sec after the bars reached the top line. Participants had to execute the choice response before the end of the trial but not sooner than 0.250 sec. before the bars reached the top line. Feedback was presented at the end of each trial, and indicated how much had been won/lost and the current balance. The feedback screen was replaced by a blank screen after 2.5 sec and the following trial started after a further 0.5 sec. We originally developed this task to examine stopping and gambling within a single block (see above). Timing of events, the rise of the bars, and the height manipulation were dictated by these stop-related factors; for example, we introduced moving bars to ensure an optimal stop-signal delay (see ref. 18, for a discussion). Similarly, we used a single manual response on each trial to manipulate response inhibition. In order to allow cross-experiment comparisons, we decided to use the same task across experiments.

On each trial, participants could win or lose points. The exact amount depended on the stake (low, medium, or high). In Experiment 1, amounts [with *p(win)*] participants could win in the low-stake condition were: 112 (0.15), 64 (0.27), 32 (0.39),16 (0.51), 6 (0.63), 2 (0.75). In Experiment 2, amounts and p(win) were: 64 (0.2), 32 (0.25), 16 (0.325), 8 (0.47), 4 (0.605), 2 (0.875). On losses, they lost half the chosen amount. Amounts decreased exponentially to make the higher amounts more attractive. We changed probabilities and amounts in Experiment 2 to ensure that expected value was the same for the three lower amounts (consequently, selecting the lowest amount was not disadvantageous). For medium stakes, all amounts were x 2; for high stakes, amounts were x 4. Stakes and the left-right order of the amounts varied pseudo-randomly from trial to trial. The starting balance was 2500 points. In Experiment 1, the total amount won was converted to money at the end of the study (1000 points = £1), whereas in Experiment 2 participants played for points only; this change in pay-off structure was motivated by practical considerations because we could not let undergraduate students gamble for real money. Playing for points is common in the literature [29], [30] and pilot work in our lab had shown that we could replicate the findings observed in Experiment 1 of Verbruggen et al. [18] with a points-only version of the stop-gambling task.

### Data Analyses

For each participant, we calculated a ‘*betting score*’ by taking the average of all choices (Range: 1–6). Choice 1 corresponded to the smallest amount with the highest p(win); choice 6 was the highest amount with the lowest p(win). Consequently, a higher betting score indicated that participants preferred higher amounts with a lower probability of winning.

The analyses focused mostly on the test phase (but see Tables 2 and 3 for an overview of the training data). Test trials with a response that was not part of the response set, with an anticipatory response, and trials without a response were excluded; trials that followed such trials were also excluded. Finally, the first trial of the experiment was also excluded. In Experiments 1 and 2, 3.9% and 4.2% of the trials were excluded, respectively. After exclusion, we calculated mean betting score and average choice latency as a function of group.

**Table 2:** Behavioural data of training sessions for Experiments 1 and 2.

			Experiment 1		Experiment 2				
					Session 1		Session 2		t-test
Group	Trial	DV	Mean	SD	Mean	SD	Mean	SD	
Stop	Nosignal	RT	591	100	890	205	867	240	1.084
		p(acc)	0.975	0.016	0.976	0.026	0.983	0.020	1.214
		p(miss)	0.012	0.019	0.036	0.031	0.017	0.018	2.546
	Signal	p(resp)	0.496	0.007	0.484	0.017	0.488	0.022	1.683
		SOA	336	94	654	224	649	253	0.235
		SSRT	233	31	225	35	212	29	1.789
Double	Nosignal	RT	495	59	485	64	434	56	6.973
		p(acc)	0.955	0.028	0.958	0.032	0.954	0.037	0.440
		p(miss)	0.003	0.004	0.002	0.004	0.000	0.001	
	Signal	RT 1	522	69	484	63	432	54	6.531
		RT 2	533	72	453	61	376	44	10.002
		SOA	261	54	457	60	415	52	5.308
		p(miss)	0.973	0.033	0.970	0.029	0.990	0.016	3.486

T-tests for Experiment 2 indicate whether the differences between Session 1 and 2 were reliable (t-values larger than the critical t-value with< = .05 are underlined). All latencies are in ms. DV = dependent variable. RT = reaction time in ms (RT 1 = RT for first response; RT 2 = RT for second response on double-signal trials). Stop-signal reaction times (SSRT) were estimated using the integration method [38].

**Table 3:** Overview of analyses of variance, comparing no-signal performance in the double-response and stop groups.

Experiment	DV	IV	F	df	MSE	p
1	RT	Condition	19.79	1,57	6777	<.001
	Acc	Condition	11.40	1,57	0.0005	<.01
2	RT	Condition	83.53	1,46	50467	<.001
		Session	10.73	1,46	3051	<.01
		C × S	1.50	1,46	3051	0.27
	Acc	Condition	10.67	1,46	0.001	<.01
		Session	0.14	1,46	0.0005	0.71
		CxS	1.17	1,46	0.0005	0.28

DV = dependent variable; IV = independent variable. RT = reaction time; Acc = accuracy.

All data processing and analyses were completed using R (R Development Core Team, 2008). Raw data files and R scripts used for the analyses are deposited on the Open Research Exeter data repository (http://hdl.handle.net/10871/4461). Because we predicted that betting scores would be lower in the stop group than in the double-response group due to a ‘cautiousness transfer’ [18], we analysed average betting scores with one-tailed Welch t-tests. When the difference between means is in the predicted direction, the p-value for the one-tailed t-test = 0.5*p(two-tailed); but when the difference is in the opposite direction, p(one-tailed) = 1 - 0.5*p(two-tailed). We also calculated Bayes factors for the betting scores. A Bayes factor compares two hypotheses; in this study these are: the hypothesis that stop training induces cautiousness when gambling (the experimental hypothesis) and the null hypothesis (i.e. no cautiousness induced). Bayes factors vary between 0 and infinity with values of less than.33 indicating support for the null hypothesis and values greater than 3 indicating support for the alternative [31]. Following Dienes [32], we used a half-normal distribution with a standard deviation of.42, which corresponds to the numerical difference in betting scores between stop and double-response groups in Experiment 3 of Verbruggen et al. [18]. The half-normal distribution was most appropriate here because it assumes that smaller effects than in our original study (which included a 2-hour rather than 24-hour delay between training and test) are more probable. We calculated the Bayes factors using the R-version of Zoltan Dienes’ Bayes calculator (http://www.lifesci.sussex.ac.uk/home/Zoltan_Dienes/inference/bayes_factor.swf).

## Results

### Experiment 1

All relevant training data are presented in Table 2. We excluded one participant in the double-response group from further analyses because their accuracy on no-signal trials was below 85%.

In the training phase, reaction times were longer and accuracy was higher on no-signal trials in the stop group than in the double-response group. This is consistent with our previous findings [18], [28], [33], and suggests that participants in the stop group were more cautious when executing their responses on no-signal trials. These conclusions were supported by one-way ANOVAs (Table 3).

**Figure 2:**
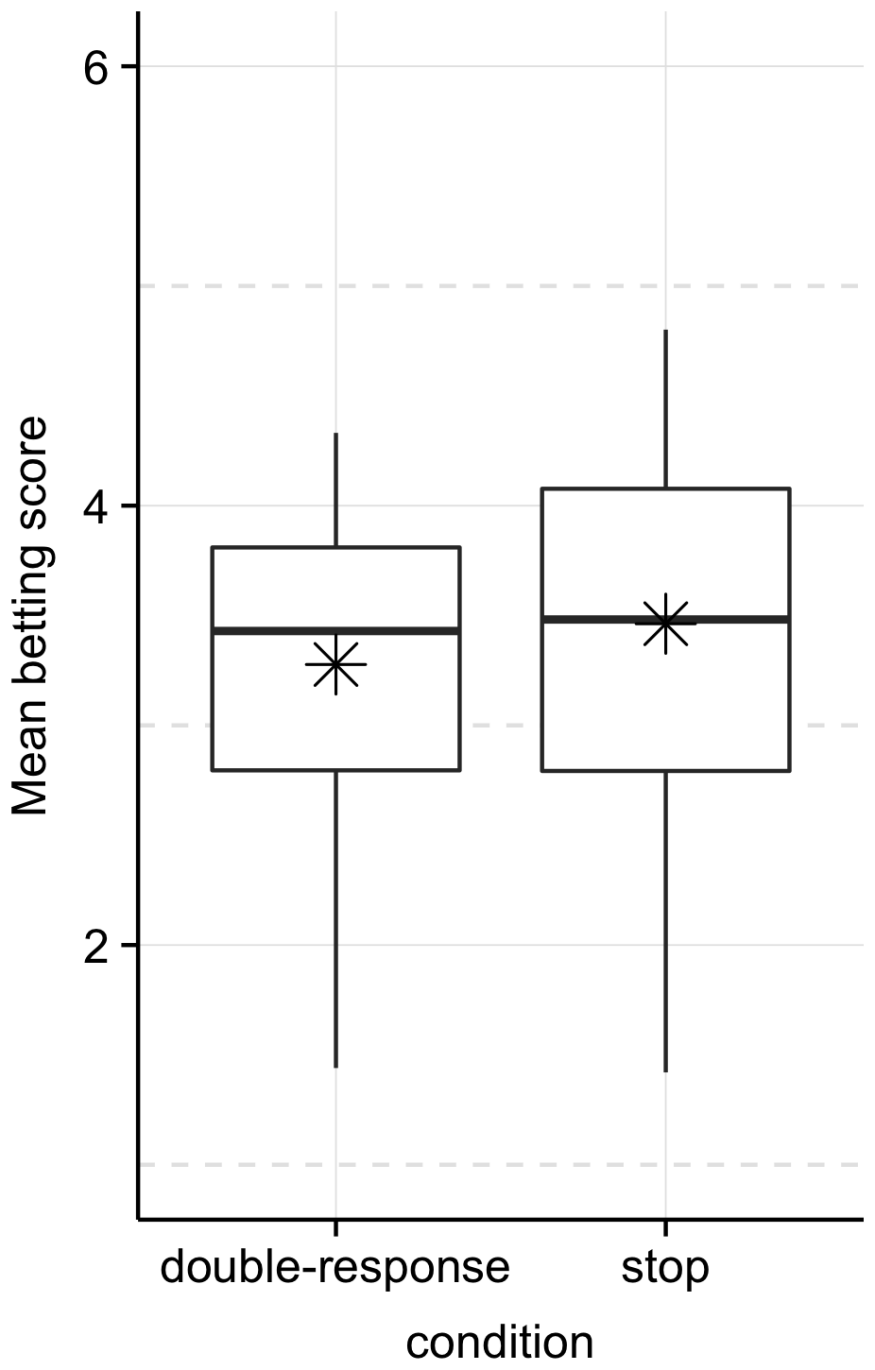
Average betting scores for the double-response and stop groups in Experiment 1 (minimum = 1; maximum = 6). In each box, the horizontal solid lines show the medians and the asterisks show the means. The upper and lower “hinges” correspond to the first and third quartiles. The vertical lines at their respective end points capture the location of extreme values. There were no outliers (i.e. values exceeding the interquartile distance by more than 1.5).

For the test phase, we compared betting scores in the stop- and double-response groups (Figure 2). Unlike in Verbruggen et al. [18], betting scores were not lower in the stop group (*M* = 3.46; *SD* = 0.84) than in the double-response group (*M* = 3.28; *SD* = .71), *t*(55.962) = 0.919, *p* = 0.819. Thus, there was no detectable transfer of cautiousness between sessions when the delay between them was 24 hours. Box-plots indicated that this was unlikely to be due to a few outlying subjects (Figure 2), and a Bayes factor (see Materials and Methods for explanation) indicated the data provided strong support for the null, relative to the cautiousness-transfer hypothesis, *B* = 0.249. This indicates that the cautiousness transfer was indeed absent. Finally, choice latencies were also comparable for the stop group (*M* = 1514 ms; SD = 92) and the double-response group (*M* = 1522 ms; SD = 92), *t(56.943)* = -0.339, *p = *0.632.Thus, the results of Experiment 1 suggested that completing a single session of stop training did not influence gambling behaviour after a 24 hour delay.

### Experiment 2

All relevant training data are again presented in Tables 1 and 2. Three subjects from the double-response group were excluded because the percentage of missed double-responses was higher than 15%; one participant in the double-response group was excluded because accuracy on no-signal trials was below 85%; and two participants from the stop group were excluded because the percentage of missed responses on no-signal trials was larger than 15%. After exclusion, there were 24 participants in each group.

Consistent with the results of Experiment 1 and our previous studies, we found that response latencies were longer and accuracy rates higher in the stop group than in the double-response group (see Tables 2 & 3). Again, this demonstrates that participants become more cautious when they are instructed to stop their responses occasionally.

**Figure 3:**
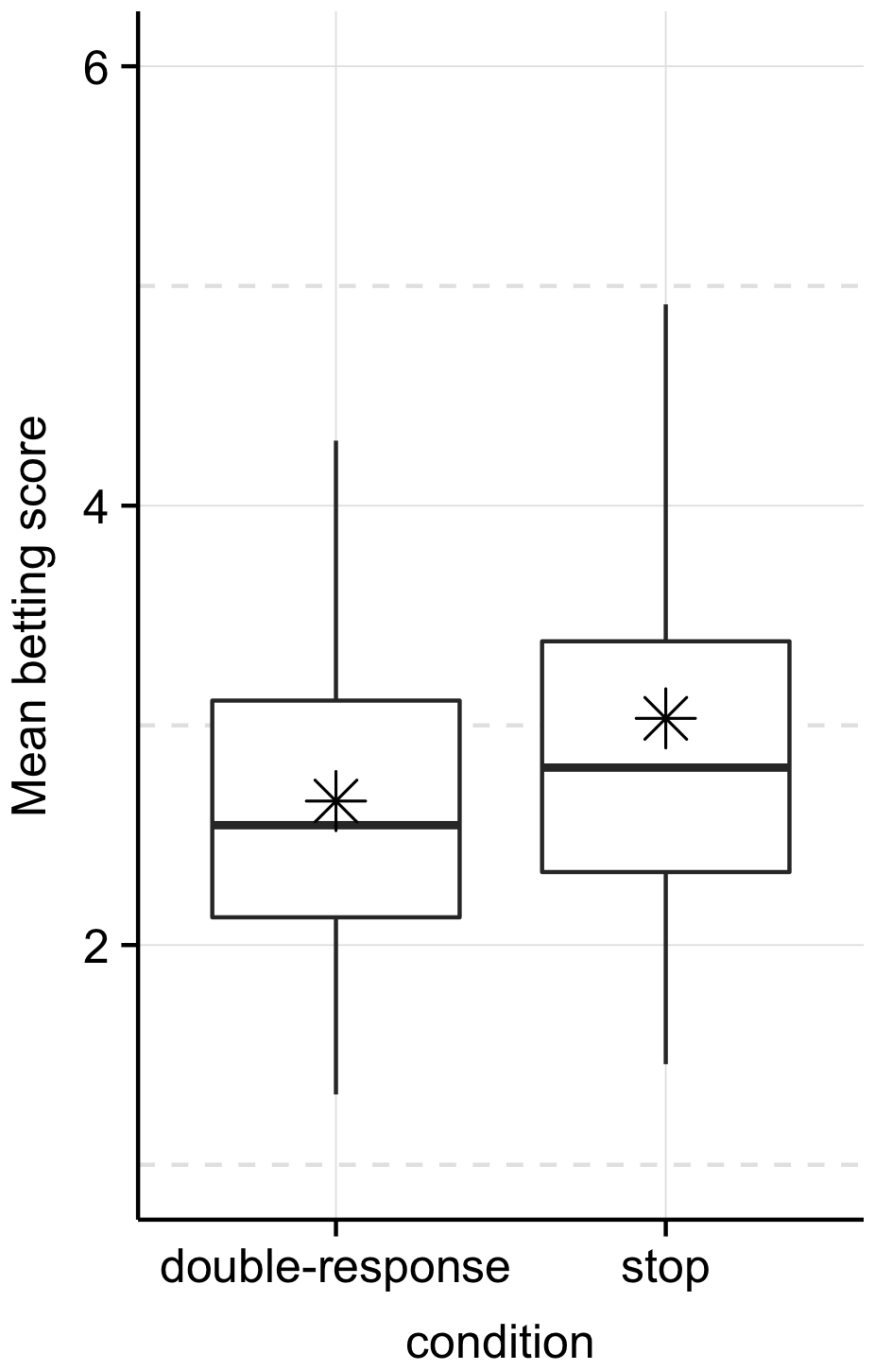
Average betting scores for the double-response and stop groups in Experiment 2 (minimum = 1; maximum = 6). In each box, the horizontal solid line shows the median and the asterisk shows the mean. The upper and lower “hinges” correspond to the first and third quartiles. The vertical lines at their respective end points capture the location of extreme values. There were no outliers (i.e. values exceeding the interquartile distance by more than 1.5).

We examined whether two days of stop training influenced gambling behaviour on the third day by comparing betting scores (Figure 3) and choice latencies for the stop and double-response groups. Again, cautiousness did not transfer between sessions, as participants in the stop group (*M* = 3.03; *SD* = .92) did not develop a stronger preference for the lower amounts with higher probability of winning compared with participants in the double-response group (*M* = 2.66; *SD* = 0.82), *t(45.454)* = −1.492, p = 0.929. The Bayes factor for betting scores also indicated strong support for the null hypothesis, which states that cautiousness does not transfer between training and test phases, *B* = 0.23. Finally, choice latencies also suggested that there was no training effect (stop = 1456 ms, *SD* = 66; double-response = 1470 ms, *SD* = 47), *t(41.703)* = 0.832, *p* = 0.795.

### Correlation between Stopping and Gambling

Previous studies found that poor response inhibition predicted risky decision-making in the Iowa Gambling Task [34]–[36]. The link between stopping and gambling or risky decision-making is also supported by the finding that response inhibition is impaired in problem gamblers (see above). Here we explored whether stop performance and betting scores in our gambling task correlated. In order to have sufficient power, we combined the data of Experiments 1 and 2. We estimated the covert latency of the stop process (stop-signal reaction time or SSRT) with the integration method [37], [38], which assumes that the finishing time of the stop process corresponds to the *n^th^* RT, where *n* = the number of RTs in the RT distribution multiplied by the overall p(respond|signal); SSRT can then be estimated by subtracting the mean SOA from the *n^th^* RT. In Experiment 2, we estimated SSRT for each session separately, and then took the average.

**Figure 4:**
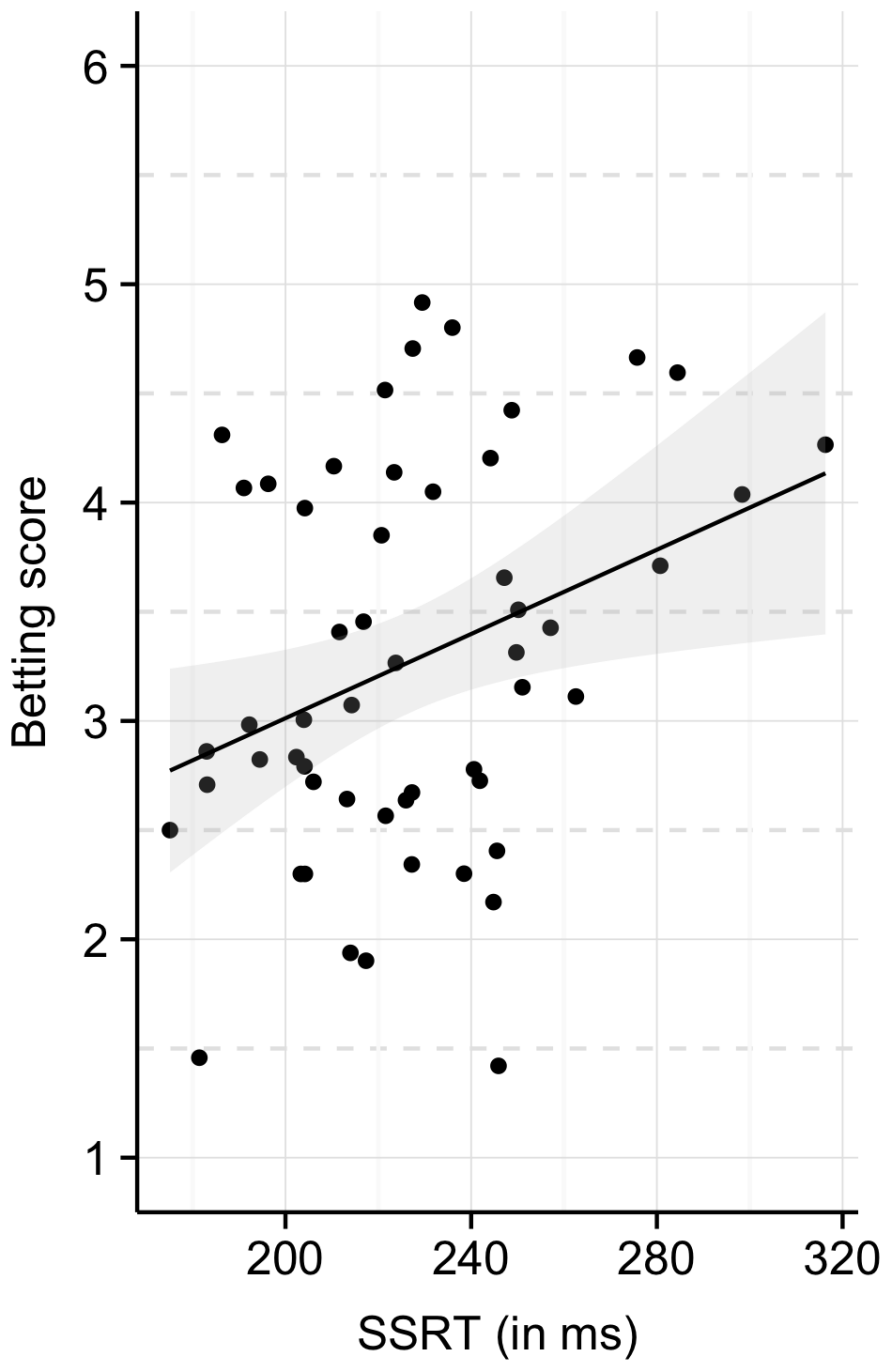
Correlation between the betting score and stop-signal reaction time (SSRT).

We found a statistically significant positive correlation (*r* = .32, *p* = .017) between SSRT and betting scores: subjects who tended to exhibit longer SSRTs in the training phase also preferred higher amounts with a lower probability of winning in the test phase (see Figure 4). This is consistent with previous findings in the Iowa Gambling Task (see above). Furthermore, it suggests that, despite the absence of a transfer of cautiousness between the two tasks, there is a link between performance in the stop-signal task and decision-making in the gambling task.

## Discussion

We have recently demonstrated that participants preferred lower amounts with a higher probability of winning in situations where they expect to stop an initiated motor response (Experiment 1, ref. 18). Furthermore, we have found that training people, even briefly, in controlling their own motor actions can induce cautious decision-making for up to two hours afterwards (Experiments 2–3, ref. 18). These experiments suggest that executive motor control can transfer to other decision-making domains, in this case monetary gambling.

In the present study, we further explored the potential of using the stop task to make people more cautious when making decisions. Compared with Experiment 3 of Verbruggen et al. [18], we increased the delay between training and test phases to 24 hours. In two experiments, participants performed either a stop task or double-response task during training. A comparison between the two groups showed that the stop group was more cautious during the training phase than the double-response group. However, in both experiments, this motor cautiousness did not transfer to the test phase in which the participants performed the same gambling task as in our previous gambling studies. We calculated Bayes factors for both experiments separately, and the combined Bayes factor was 0.06 (0.249 * 0.234). Thus, the data of the two studies combined provide ‘very strong’ evidence [31] for the hypothesis that cautiousness does not transfer from the stop task to the gambling task when the delay between the two phases is increased to 24 hours. Although sleep plays in important role in declarative and procedural memory consolidation [39], the results of the present study suggest that stop training does not necessarily benefit from sleep.

One possibility is that the amount of training of general inhibitory skills that caused a transfer effect when the delay was short was not strong enough to illicit changes in behaviour later in time (although doubling the amount of training did not alter the results). Guerrieri and colleagues [40] recently reached a similar conclusion. They examined to what extent performing an inhibitory control task influenced subsequent eating behaviour, as several studies have demonstrated that eating disorders correlate with impairments in inhibitory control [41]–[44]. They found that participants who had to respond quickly (the impulsivity group) tended to eat more during a subsequent bogus taste test compared with a group who had to stop more and a neutral baseline control group; importantly, the stop group and baseline group did not differ. Based on these findings, the authors concluded that effects of inhibition training may be weaker than originally assumed.

Alternatively, the results observed in Verbruggen et al. [18] may not have been caused by *training* of inhibitory control. Based on our previous findings, it is tempting to conclude that performing a short stop task can strengthen the inhibition control network. However, an alternative explanation is that our previous results were caused by a task carry-over effect. When participants perform a stop-signal task, they often make proactive control adjustments to balance between impulsive responses, which are difficult to suppress, and slow responses, which can be stopped more easily [28]. Such control adjustments may involve activating the stop goal in advance and increasing decision thresholds in the choice task [28], [45], [46]. Participants may have adopted a similar ‘proactive’ control strategy when performing the gambling task. For instance, keeping the overall long-term goal activated (i.e. ‘have as many points as possible at the end of the experiment’) could result in a more consistent choice pattern; higher decision thresholds would also result in a more consistent choice pattern. It seems likely that such a transfer is more likely to occur when the stop task was executed recently.

A potential limitation of this study is that this is a cross-study comparison. However, the only difference between the design of Experiment 1 of the present study and the design of Experiments 2–3 in Verbruggen et al. [18] was the delay between training and test. Thus, it seems unlikely that differences between the present study and our previous work are caused by differences in design other than the delay manipulation. Nevertheless, it is possible that subtle differences between participants had an influence. People with poor inhibitory control or problem gamblers may benefit more from doing a stop-signal task than people who never gamble; indeed, developmental studies have demonstrated that children with poor executive control benefited the most from executive control training [22], [47], [48]. We did not have a baseline measurement of gambling or inhibition in our task, so we could not explore this issue in the current study. Other individual differences may also have contributed. For example, Colzatto and colleagues have found that inter-individual genetic variability modulated transfer between a training and a test task [49]. Thus, an important avenue for future research is to determine how individual differences modulate transfer effects.

Another potential avenue for future research is exploring whether gambling-related stop learning induces longer-lasting effects. We have previously demonstrated that executive control processes such as stopping can also be triggered in a bottom-up fashion by the retrieval of previously acquired stimulus-stop associations [50]. Other studies have already demonstrated that such stimulus-stop associations can influence behaviour outside the lab. For example, it was found that a consistent pairing of alcohol-pictures to stopping reduced the subsequent weekly alcohol intake [51] or consumption in the lab [52]. Similarly encouraging stimulus-specific training effects have been found in food studies [53], [54]. However, future research is required to explore the potential of such training regimes for various impulse-control disorders.
